# Complete Genome Sequence of Pseudomonas sp. Strain MM213, an Isolate from a Brookside in Bielefeld, Germany

**DOI:** 10.1128/mra.00866-21

**Published:** 2022-01-06

**Authors:** Bart Verwaaijen, Özgülen Cevahir, Fabian Hitz, Jacqueline Römmich, Donat Wulf

**Affiliations:** a Computational Biology, Faculty of Biology, CeBiTec, Bielefeld University, Bielefeld, Germany; b Biology, Bielefeld University, Bielefeld, Germany; c Molecular Biotechnology, Bielefeld University, Bielefeld, Germany; d Graduate School DILS, Bielefeld Institute for Bioinformatics Infrastructure, Bielefeld University, Bielefeld, Germany; University of Arizona

## Abstract

Here, we report the genome sequence of Pseudomonas sp. strain MM213, isolated from brookside soil in Bielefeld, Germany. The genome is complete and consists of 6,746,355 bp, with a GC content of 59.4% and 6,145 predicted protein-coding sequences. Pseudomonas sp. strain MM213 is part of the Pseudomonas mandelii group.

## ANNOUNCEMENT

The genus Pseudomonas is composed of Gram-negative, rod-shaped gammaproteobacteria and is the most diverse bacterial taxon known to date, with 242 validated species (1). Members of the genus Pseudomonas inhabit a wide variety of environments, possess great metabolic variety, and have the potential for adaptation to unstable environmental conditions (1).

The soil sample, which was claylike and wet without any vegetation present, was obtained from a brookside in Bielefeld, Germany (52°01′45.5″N, 8°29′11.9″E), suspended in saline solution (0.9% [wt/vol] NaCl), and shaken for 5 min. The sample was taken from 5 cm beneath the ground. The suspension was filtered using a cellulose filter (REF 431015; Macherey-Nagel, Düren, Germany) and centrifuged, and the cell pellet was resuspended in fresh saline solution. Various dilutions were plated on agar medium (1.5% agar, 1% peptone from soy, 0.3% NaCl, 0.1% sucrose, 0.1% cellulose, 0.1% xylan, 0.1% chitin, and 0.05% Tris-HCl) and incubated at 28°C for 1 week. Colonies formed on agar plates were yellow. A single colony was picked for DNA isolation.

The NucleoSpin microbial DNA minikit for DNA from microorganisms (REF 740235; Macherey-Nagel) with optional RNA digestion was used to isolate the genomic DNA. With this DNA, a library was constructed with the native barcoding kit (EXP-NBD104; Oxford Nanopore Technologies, Oxford, UK) according to the manufacturer’s specifications and sequenced with an R9.4.1 flow cell (Oxford Nanopore Technologies). All software was used with default parameters unless otherwise specified. DNA sequences were called live using the Super accuracy base-calling model (MinKNOW v1.4.3; Oxford Nanopore Technologies) on a GridION system. Adapters were trimmed using Porechop v0.2.4 (2). In total, 134,282 reads with 1.1 billion bases, with an *N*_50_ value of 14,732 bp, were sequenced; 50% of the data was trimmed with Filtlong v0.20 (3). The genome was assembled with Canu v2.1.1 (4) and polished with Racon v1.4.3 (5), followed by a second polishing with Medaka v1.4.3 (Oxford Nanopore Technologies). The genome had a coverage of 89×. The bacterial strain was identified using the BLAST function of the Type (Strain) Genome Server (TYGS) (6). Completeness was examined with BUSCO v5.1.2 (7). Genes were predicted with Prodigal v2.6.2 (8). The metabolic pathways were examined with KAAS (9). Potential secondary metabolite gene clusters were identified using antiSMASH (10).

**FIG 1:**
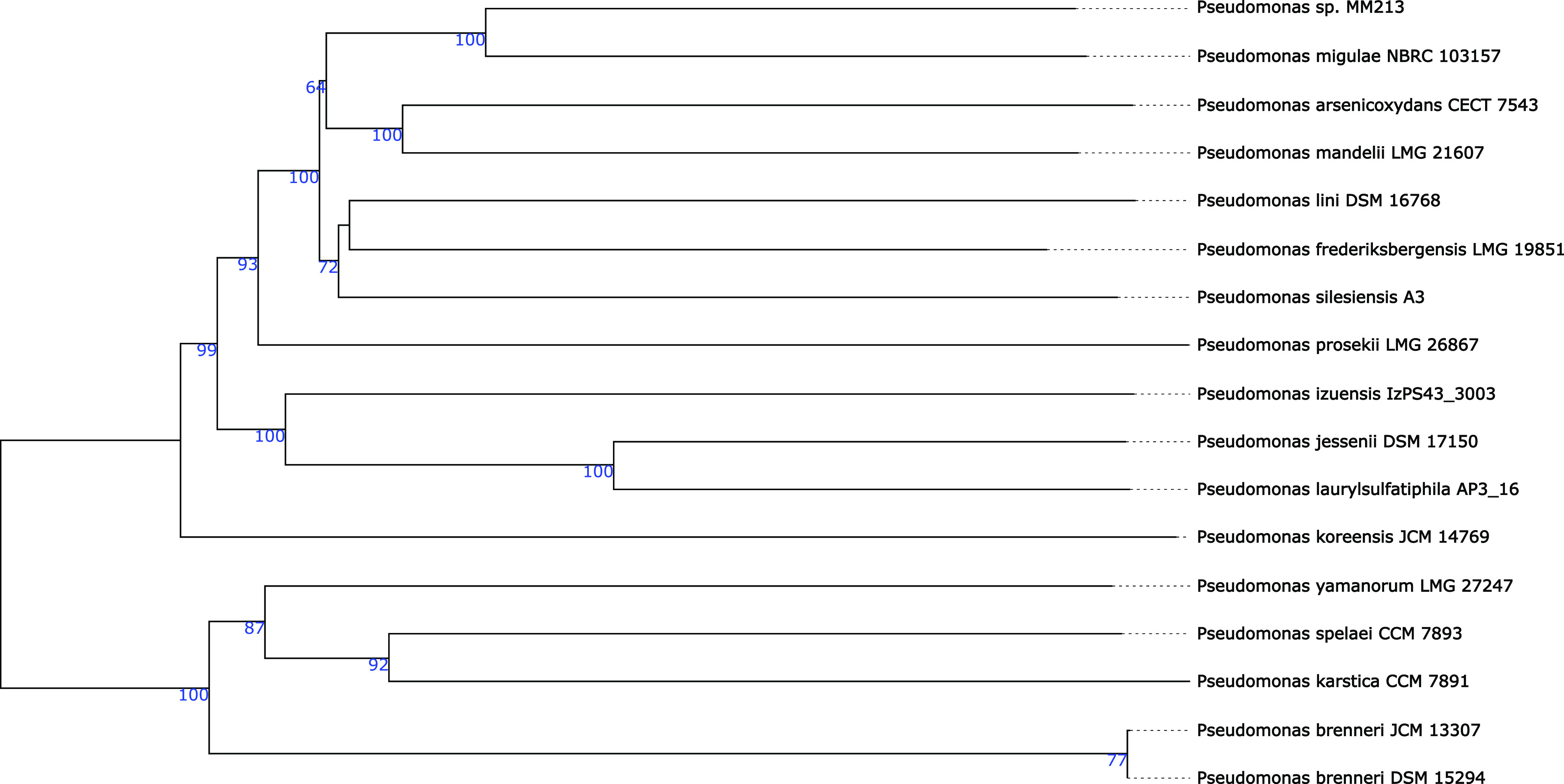
Genome BLAST Distance Phylogeny (GBDP) tree (6). A phylogenetic tree was constructed with TYGS (6). The tree represents only the Pseudomonas strains most closely related to the described isolate, Pseudomonas sp. strain MM213 (6). The bootstrap support values from 100 replications are marked in blue at each branch point. The branch lengths are scaled in terms of GBDP distance formula *d*_5_. The numbers above the branches are GBDP pseudo-bootstrap support values of >60% from 100 replications. The tree was rooted at the midpoint. The numbers at the end of each branch represent the strain numbers for each species.

Pseudomonas sp. strain MM213 phylogenetically groups within the subgroup Pseudomonas mandelii and in the major group Pseudomonas fluorescens (11) (Fig. 1). The most closely related known organism is Pseudomonas migulae NBRC 103157 (GenBank assembly accession number GCA_002091715.1), with a *d*_4_ digital DNA-DNA hybridization (dDDH) similarity of 46.4% (6). A member of the subgroup P. mandelii is a cold-adapted, nonhalophilic bacterium that can grow at 4°C but does not grow at 37°C (12). P. mandelii strains do occur in mineral waters and agricultural fields (13). The assembly had a completeness of 96% (Table 1).

**TABLE 1:** BUSCO results for Pseudomonas sp. strain MM213

BUSCO parameter	Result (%)
Completeness	96
Single copy	95.7
Duplicated	0.3
Fragmented	0.3
Missing	3.7

Pseudomonas sp. strain MM213 has the potential to produce secondary metabolites such as vitamins B1 to B5, B7, and B12 (9). The genome suggests the presence of flagella and benzoate degradation (9).

Pseudomonas sp. strain MM213 is likely able to produce aryl polyenes, because 45% of the genes within this cluster have a significant BLAST hit to the gene cluster from Aliivibrio fischeri ES114 (GenBank assembly accession number GCA_000985635.1) (10, 14). Aryl polyenes are yellow carotenoid-like pigments that have been found to be antioxidants and protection against reactive oxygen species (15).

### Data availability.

The genome sequence of Pseudomonas sp. strain MM213 has been deposited in NCBI GenBank under accession number CP081943. The NCBI Sequence Read Archive (SRA) accession number for the raw reads is SRR15533117.

## References

[1] Silby MW, Winstanley C, Godfrey SA, Levy SB, Jackson RW. 2011. *Pseudomonas* genomes: diverse and adaptable. FEMS Microbiol Rev 35:652–680. doi:10.1111/j.1574-6976.2011.00269.x. 21361996

[2] Wick RR, Judd LM, Gorrie CL, Holt KE. 2017. Completing bacterial genome assemblies with multiplex MinION sequencing. Microb Genom 3:e000132. doi:10.1099/mgen.0.000132. 29177090 PMC5695209

[3] Wick R. 2021. Filtlong. https://github.com/rrwick/Filtlong.

[4] Koren S, Walenz BP, Berlin K, Miller JR, Bergman NH, Phillippy AM. 2017. Canu: scalable and accurate long-read assembly via adaptive *k*-mer weighting and repeat separation. Genome Res 27:722–736. doi:10.1101/gr.215087.116. 28298431 PMC5411767

[5] Vaser R, Sović I, Nagarajan N, Šikić M. 2017. Fast and accurate de novo genome assembly from long uncorrected reads. Genome Res 27:737–746. doi:10.1101/gr.214270.116. 28100585 PMC5411768

[6] Meier-Kolthoff JP, Göker M. 2019. TYGS is an automated high-throughput platform for state-of-the-art genome-based taxonomy. Nat Commun 10:2182. doi:10.1038/s41467-019-10210-3. 31097708 PMC6522516

[7] Manni M, Berkeley MR, Seppey M, Simão FA, Zdobnov EM. 2021. BUSCO update: novel and streamlined workflows along with broader and deeper phylogenetic coverage for scoring of eukaryotic, prokaryotic, and viral genomes. Mol Biol Evol 38:4647–4654. doi:10.1093/molbev/msab199. 34320186 PMC8476166

[8] Hyatt D, Chen G-L, Locascio PF, Land ML, Larimer FW, Hauser LJ. 2010. Prodigal: prokaryotic gene recognition and translation initiation site identification. BMC Bioinformatics 11:119. doi:10.1186/1471-2105-11-119. 20211023 PMC2848648

[9] Moriya Y, Itoh M, Okuda S, Yoshizawa A, Kanehisa M. 2007. KAAS: an automatic genome annotation and pathway reconstruction server. Nucleic Acids Res 35:W182–W185. doi:10.1093/nar/gkm321. 17526522 PMC1933193

[10] Blin K, Shaw S, Kloosterman AM, Charlop-Powers Z, van Wezel GP, Medema MH, Weber T. 2021. antiSMASH 6.0: improving cluster detection and comparison capabilities. Nucleic Acids Res 49:W29–W35. doi:10.1093/nar/gkab335. 33978755 PMC8262755

[11] Garrido-Sanz D, Meier-Kolthoff JP, Göker M, Martín M, Rivilla R, Redondo-Nieto M. 2016. Genomic and genetic diversity within the *Pseudomonas fluorescens* complex. PLoS One 11:e0150183. doi:10.1371/journal.pone.0150183. 26915094 PMC4767706

[12] Jang SH, Kim J, Kim J, Hong S, Lee C. 2012. Genome sequence of cold-adapted *Pseudomonas mandelii* strain JR-1. J Bacteriol 194:3263. doi:10.1128/JB.00517-12. 22628497 PMC3370865

[13] Weon H-Y, Dungan RS, Kwon S-W, Kim J-S. 2007. The phylogeny of fluorescent pseudomonads in an unflooded rice paddy soil. Ann Microbiol 57:299–306. doi:10.1007/BF03175064.

[14] Altschul SF, Gish W, Miller W, Myers EW, Lipman DJ. 1990. Basic local alignment search tool. J Mol Biol 215:403–410. doi:10.1016/S0022-2836(05)80360-2. 2231712

[15] Schöner TA, Gassel S, Osawa A, Tobias NJ, Okuno Y, Sakakibara Y, Shindo K, Sandmann G, Bode HB. 2016. Aryl polyenes, a highly abundant class of bacterial natural products, are functionally related to antioxidative carotenoids. Chembiochem 17:247–253. doi:10.1002/cbic.201500474. 26629877

